# Acoustic and visual traits predict species nuclearity in Neotropical mixed-species bird flocks

**DOI:** 10.1007/s00442-026-05900-x

**Published:** 2026-05-11

**Authors:** Vicente García-Navas, Carlos Martínez-Nuñez

**Affiliations:** 1 Department of Ecology and Evolution, Estación Biológica de Doñana EBD (CSIC), Avenida, Américo Vespucio, 26, E-41092 Seville, Spain; 2 Department of Evolutionary Biology and Environmental Studies, University of Zurich, Zurich, Switzerland

**Keywords:** Centrality, Flocking propensity, Heterospecific sociality, Neotropics, Network, SMF

## Abstract

Mixed-species bird flocks constitute a striking example of heterospecific sociality, offering participants potential benefits such as enhanced foraging efficiency and reduced predation risk. Yet, not all species join these flocks with the same frequency. Moreover, beyond simply participating, species may play distinct roles within flock networks. While some bird species may exhibit high nuclearity and occupy a central position in flock formation, cohesion, and dynamics (i.e., social hubs), others are merely peripheral followers. Understanding how phenotypic and ecological traits relate to both flocking propensity and network position can provide key insights into the drivers of interspecific social behavior in birds. Using a comprehensive dataset of over 3,000 flocks, we applied network theory to explore the relationship between species-level network metrics (connectivity, strength, and closeness), flocking propensity, and key phenotypic traits (residual eye size, beak shape, maximum song frequency, and plumage coloration) in Neotropical birds. Bayesian phylogenetic models revealed that flocking propensity was positively associated with maximum song frequency and carotenoid-based coloration, and negatively associated with residual eye size. In addition, the extent of white in plumage significantly predicted species centrality. This is the first study to evaluate whether acoustic and visual traits predict both flocking propensity and a species’ structural role within flock networks. These findings, along with the moderate phylogenetic signal detected in network metrics, suggest that the evolution of heterospecific sociality may involve distinct morphological and sensory adaptations.

**Supplementary Information:**

The online version contains supplementary material available at 10.1007/s00442-026-05900-x.

## Introduction

Heterospecific foraging associations are a widespread phenomenon in nature, documented across a wide range of taxa, including invertebrates, fishes, birds, and mammals (Goodale et al. 2017; 2020). By forming coalitions or flocks, species can gain benefits such as reduced predation risk and/or increased foraging efficiency (Sridhar et al. 2009; Sridhar and Guttal, 2018; Beauchamp and Krams 2023; Carlson et al. 2023). Mixed-species animal groups thus exemplify the importance of facilitation processes in structuring community assembly at small spatial scales (Mammides et al. 2018; Sridhar and Guttal, 2018, 2015). However, some studies—mainly from the Neotropical region—have suggested that competition may be a stronger driver than facilitation in flock formation, leading to ‘checkerboard’ patterns among species (Colorado and Rodewald 2015). These interspecific interactions—revealing either preferential assortment or avoidance of specific flock mates—generate a network of associations at the population level, where species are connected in structured ways. Analyzing these networks can offer valuable insights into the assemblage rules of such micro-communities (Sridhar et al. 2013).

In this context, network analysis—a tool derived from graph theory—can be used to detect internal substructures, such as tightly connected clusters of nodes, based on observed associations (Farine et al. 2012; Shizuka and Farine 2012). In heterospecific social aggregations such as mixed-species bird flocks, these modules may emerge from non-random biotic interactions and species-specific microhabitat preferences within the network, which consists of nodes (representing species) connected by edges (representing observed co-occurrences in flocks) (Mokross et al. 2014; Borah et al. 2018; Bangal et al. 2022). A network framework thus allows us to assess the role of each species in the structure and stability of the social system it belongs to. This is relevant because not all species within a group play the same role. Some bird species are frequently encountered across habitats, show a high propensity to flock, and/or show specific behaviours within the flock, and are therefore considered leader or “nuclear” species (Goodale et al. 2020; Bangal and Sridhar, 2023). These species often attract other participants (attendant or satellite species) and typically lead flock movements, playing a central role in the formation and cohesion of these heterospecific social groups.

By examining the connections and participation patterns of each species across flocks (i.e., the number and identity of flocks it joins), it is possible to assess its position within the metanetwork and thus determine its degree of nuclearity (centrality sensu Schoch 2018). This quantitative approach provides a better understanding of the relationship between a species’ functional importance in interactions and its traits, and represents an improvement over the traditional method of classifying participants in multi-species groups into two categories—nuclear and attendant species—based solely on participation frequency (Srinivasan et al. 2010). A priori, species with a higher propensity to flock are more likely to play a nuclear role in flock co-occurrence networks than those with a much lower likelihood of engaging in this behavior. In this context, a few studies have examined the ecological and phenotypic predictors of mixed-species flocking propensity, using it as a proxy for nuclearity (Thiollay and Jullien 1998; Martínez et al. 2016). For instance, Beauchamp and Mangini (2024) recently reported that larger and nectarivore species were less likely to join mixed-species flocks after controlling for phylogeny. However, the relationship between flocking propensity and nuclearity is not necessarily linear or deterministic. A species may exhibit high flocking propensity by participating frequently in flocks, yet consistently associate with the same limited set of species, resulting in a low or moderate connectivity and centrality. However, given the same flocking propensity, a species that interacts with a highly diverse set of species will have comparatively higher centrality, more connections (higher degree), and a relatively lower strength in co-occurrence matrices, as it acts as a social hub. Therefore, while a positive correlation is anticipated, flocking propensity and nuclearity capture distinct aspects of social behavior: the former quantifies a general tendency to participate in mixed-species groups, whereas the latter reflects the centrality of species and the diversity of species-level associations formed during such interactions.

In addition, few studies to date have investigated whether acoustic and visual traits predict species’ flocking propensity or their topological importance in network structure (see e.g., Pagani-Núñez et al. 2018 for an exception). If a species’ tendency to join or initiate flocks reflects its potential nuclearity, one might hypothesize that species playing roles such as leading the group and/or acting as sentinels to alert others to potential threats may exhibit distinctive acoustic and visual attributes (e.g., flash plumage signals; Benedict et al. 2025). These traits could help attract and maintain flock members, promoting group cohesion during movement or foraging. In this vein, some local studies have proposed that nuclear species are louder and more conspicuously colored than satellite species (reviewed in Bangal and Sridhar 2023). Nevertheless, this hypothesis has yet to be rigorously tested at a broader, macroevolutionary scale.

Although several authors have applied network theory to the study of mixed-species bird flocks (e.g., Montaño-Centellas et al. 2023), most have focused on variation in network-level metrics along environmental gradients. For example, Montaño-Centellas and Garitano-Zavala (2015) examined the effect of elevation on the structure of social networks in Andean bird flocks along a transect ranging from 2,000 to 3,550 m a.s.l. They found that networks at higher elevations were less modular, had lower strength, and exhibited less skewed degree distributions than those at lower elevations. In contrast, the employment of species-level metrics is much scarcer, even though these can be useful for linking species traits to their social roles and influence within mixed-species flocks, offering greater insight than simple co-occurrence analysis.

In this study, we use species-level metrics from mixed-species flocks’ co-occurrence networks to examine the relationships between species’ flocking propensity, their connectivity and position within mixed-species flock networks (quantified using various network metrics), and four phenotypic traits: residual eye size, beak shape, maximum song frequency, and plumage coloration. Specifically, we test four distinct predictions. First, plumage coloration in birds plays an important role in social behaviour, and certain color patterns can facilitate visual communication within flocks, helping birds coordinate movements and activities (Strandburg-Peshkin et al. 2013). Thus, nuclear species (species with high flocking propensity, high connectivity and that occupy a central position) are expected to exhibit more conspicuous plumage patterns that facilitate flock cohesion. Second, having more eyes looking for danger is likely to be a benefit (‘many eyes’ effect; Morse 1977), so nuclear species may experience weaker selective pressure on eye size than other species. Consequently, species that frequently join mixed-species flocks may rely less on their own visual detection of predators and therefore are predicted to exhibit relatively smaller eyes. Third, acoustic communication can play an important role in flock cohesion and information transfer among heterospecifics (Bradbury and Vehrencamp 2011). Species with distinctive vocal traits may reduce acoustic overlap, facilitating signal transmission and recognition within the group. Therefore, species producing higher-frequency vocalizations are expected to show higher flocking propensity and stronger interaction patterns within the network (Pagani-Núñez et al. 2018).

Lastly, beak morphology is closely linked to food exploitation in birds, and thus represents a key functional trait shaping ecological interactions among co-existing species. In mixed-species flocks, species with different trophic niches may benefit from complementary foraging strategies, which can reduce competition and promote ecological complementarity. Species with more generalized morphological traits —such as intermediate beak shapes— may interact with a broader range of species (i.e., multiple distinct functional groups), and therefore are predicted to occupy more central positions in the network (Dehling et al. 2016).

## Material and methods

### Mixed-species bird flocks’ data

We conducted an exhaustive literature search in Scopus and Google Scholar to gather information on the composition of mixed-species bird flocks using the English and Spanish terms “mixed-species flocks”, “MSF”, and “bandos mixtos”. We restricted our search to the Neotropical region and only included studies that reported the composition of each surveyed flock (i.e., occurrence data), either in a table or in the appendices as supplementary material. In total, we compiled data on 3,458 mixed-species bird flocks from 33 studies conducted in 83 different localities across nine South American countries (Table S1). We only considered flocks composed of at least three species to ensure a minimal flock size. Across these 3,458 flocks, a total of 559 bird species were identified, after excluding those recorded in fewer than three flocks. Thus, species whose presence in such flocks is anecdotal or non-existent were excluded from our analyses and we focused on species that, to varying degrees, participate in mixed-species bird flocks. The absence of “zeros” (i.e., species that do not occur in mixed-species flocks) in our data implies that our analysis centers on the variability of traits among species that do participate in mixed-species flocking, whether frequently or infrequently (i.e., a continuum from flocking and/or ‘most nuclear’ species to occasionally flocking and/or ‘least nuclear’ species).

From this dataset, and because information on the complete local species pool was not consistently available across studies, we computed each species’ propensity to join mixed-species flocks, estimated as the proportion of flocks in which it was detected. That is, this metric therefore reflects relative differences among species in their participation in mixed-species flocks rather than the probability of joining flocks conditional on local presence. Our definition of flocking propensity thus differs from the one commonly used in prior work, which quantifies the proportion of observations in which a given species participates in mixed-species flocks (e.g., Beauchamp and Mangini 2024). In such studies, a species’ propensity to join mixed-species flocks is typically assessed from observations at a single locality (i.e., *n* = 1). Here, we employed our extensive dataset to obtain a more robust and reliable estimate of flocking propensity for each species. By using flocks as the sampling unit (instead of sightings) and pooling data across multiple localities, our metric yields a spatially generalisable estimate of joining propensity that is less confounded by species abundance, detectability, and local sampling effort than observation-based, single-site measures. *Flocking propensity* was log-transformed to reduce skewness, stabilize variance, and improve model fit.

### Species-level network metrics

We analyzed the nuclearity (centrality) of species by using an interaction network approach. This approach provides a lot of versatility and useful metrics to rank the potential of species to alter the abundances of other species (Bascompte et al. 2003). For each locality (population), flock–species networks were projected into unipartite species–species networks based on co-occurrence within flocks. The resulting networks were visualized using the ‘igraph’ package (Csardi and Nepusz 2006) (Fig. S1). Next, we computed species-level metrics related to each species’ connectivity and position within the network (degree, strength, and closeness), and then these were averaged to obtain a mean value for each species. First, we obtained the degree or number of connections (i.e., the number of species with which a species co-occurs in flocks) for each node (species) in each locality (see Mokross et al. (2014) and Montaño-Centellas (2020) for a similar approach). Since degree is sensitive to flock size, we obtained its normalized counterpart (henceforth, *connectivity*) defined as the proportion of species (nodes) it interacts with out of the total possible in the network. In addition, we computed the average *strength* of species co-occurrences in the flocks (i.e., the sum of the frequency of interspecific associations of each species; see Bascompte et al. 2006). Together with connectivity, interaction strength helps distinguish species that co-occur with many partners but show weak associations (high connectivity, low strength) from those that maintain fewer but stronger associations with specific partners (low connectivity, high strength). Lastly, we computed the average weighted closeness centrality (henceforth, *closeness*), which measures the proximity of a node to any other node in the network (i.e., its centrality), taking into account the species’ representation in the metanetwork (i.e., across the set of local networks). We used the inverse of the edge weights, such that stronger species–species associations were interpreted as shorter distances between nodes. Thus, nodes with high closeness values can rapidly affect other nodes and vice versa. Species-level metrics were averaged across the localities where each species occurred to obtain an overall estimate of its structural role. Connectivity was calculated in its normalized form (proportion of realized interactions relative to the total possible connections in the network), which accounts for differences in network size across localities and allows comparisons among networks. Species-level network metrics were obtained using the R package ‘bipartite’ (Dormann et al. 2008).

For the purposes of this study and recognizing the ambiguity of the term nuclearity (recently reviewed in Bangal and Sridhar 2023), we define a nuclear species as one that flocks frequently, has high connectivity and/or occupies a central position in the network. This quantitative approach, which allows for positioning each species along a continuum in terms of connectivity and centrality constitutes an improvement over the traditional dichotomous categorization of species into ‘nuclear’ and ‘attendant’.

### Species traits

For each species, we compiled the following traits: body mass (used as a proxy for body size), beak shape, residual eye size, maximum song frequency, and plumage coloration. Beak shape was quantified using four linear measurements (beak depth, beak width, beak length from tip to nares, and beak length from tip to culmen) obtained from Tobias et al. (2022). These measurements were size-corrected using the *phyl.resid* function in the ‘phytools’ package (Revell 2024), and subsequently used in a principal component analysis (PCA) to summarize beak morphology. The first principal component (PC1) explained 56% of the total variation and was used as a composite index of beak shape, where negative values indicate large, thick beaks and positive values represent small, thin beaks. Residual eye size was derived from a dataset of ocular measurements collected from preserved specimens by Stanley Ritland and digitalized and made available through Ausprey (2021). Specifically, we used the size-corrected axial diameter (AD). AD was strongly correlated with the maximum outer diameter of the sclerotic ring (data from Weeks et al. 2025) (*ρ* = 0.89; *p* < 0.001), supporting its validity as a proxy for eye size. However, data on AD were only available for 282 species; therefore, analyses including this variable were performed on a subset of the full dataset.

Average maximum song frequency per species was obtained from both our own measurements (*unpublished data*) and a recent global dataset (Sagar et al. 2024). Our measurements were extracted from spectrograms analyzed using Raven Pro (Cornell Lab of Ornithology), based on recordings sourced from public repositories such as Xeno-Canto (xeno-canto.org) and the Macaulay Library (macaulaylibrary.org). We aimed to characterise each species using a minimum of three recordings and a maximum of five, although this was not always possible due to the difficulty of finding audio recordings for some species with a small distribution range or low abundance. Sex information is seldom provided for archived recordings. Therefore, acoustic traits were examined at the species level and should be interpreted as reflecting variation among species rather than differences between sexes. Ideally, species would have been acoustically characterized using alarm calls, as these vocalizations serve a social rather than a sexual function, unlike songs. However, because call recordings were unavailable for some species, we relied on song frequency instead, given the strong correlation between the frequencies of songs and calls. This relationship largely arises because both vocalization types are constrained by physiological limits of the vocal apparatus and body size (own data; see also Friis et al. 2021). Since there is a negative correlation between body size/mass and song frequency (larger birds tend to vocalize at lower frequencies; PGLS: estimate = -0.23 $\pm$ 0.04, *t* = -4.99, *p* < 0.001), we size-corrected our maximum song frequency estimates and used the residuals.

Plumage coloration was characterized for both sexes using data from Delhey et al. (2023a, b), who quantified the proportion of the body covered by 12 human-visible color categories across nearly all bird species. We grouped these 12 categories into five broader classes based on the pigment (or lack of) or structural origin of the coloration: structural (blue and purple), carotenoid-based (yellow, orange, and red), melanin-based (black, grey, brown, and rufous), green, and white. For the sake of brevity, we present results only for male coloration, implicitly assuming that all flock members are male, as unfortunately, we lack data on the sex composition of the flocks. Structural and melanin-based coloration were negatively correlated ($\rho$= -0.40, *p* < 0.001), so only the latter of these two variables was included in our models.

Prior to model fitting, we examined pairwise Pearson correlations among predictor variables and calculated variance inflation factors (VIF) to assess potential multicollinearity. All *GVIF*^1/(2Df)^ values were below 2, indicating very low levels of multicollinearity.

### Bayesian phylogenetic comparative analyses

To examine the relationship between the species-level network metrics (*connectivity*, *strength*, and *closeness*), flocking propensity, and phenotypic attributes (beak shape, residual eye size, maximum song frequency, and plumage coloration) (*n* = 559 species), we fitted Bayesian phylogenetic linear models fitted using the R package ‘brms’ (Bürkner 2017). To account for phylogenetic dependence among species, we employed as a phylogenetic hypothesis a sample of 50 phylogenetic trees from the Bayesian posterior tree set provided in BirdTree (birdtree.org) based on the Ericson backbone (Jetz et al. 2012). We accounted for topological uncertainty by iteratively running each Bayesian analysis across this sample of trees. We fitted four models, each with a different index—flocking propensity, connectivity, strength, or closeness—as the response variable. Phenotypic predictors included residual eye size, beak shape, maximum song frequency, and multiple aspects of plumage coloration (structural coloration, carotenoid-based coloration, melanin-based coloration, green coloration, and presence of white). Phylogenetic covariance was included as a random intercept. All continuous variables were standardized (mean = 0, SD = 1) to facilitate effect size comparison. We ran two Markov chain Monte Carlo (MCMC) chains for 4,000 iterations each, using default priors, with a burn-in of 2,000 iterations and thinning every 50 iterations. We assessed convergence by visually inspecting trace plots and checking standard diagnostics (*Rhat*: 1,0–1,1). After obtaining posterior distributions of slope estimates from each tree-specific model, we merged them into a single posterior distribution comprising 200,000 samples (5,000 samples × 50 trees). From this combined distribution, we report posterior means as point estimates and 95% credible intervals. Alternatively, we adopted a probabilistic approach and fitted the same models using phylogenetic generalized linear models (PGLS) in ‘caper’ (Orme et al. 2013). For the sake of brevity, and since the results obtained from both approaches were quite similar, we only report here the results obtained using a Bayesian framework. We then assessed the interdependence between flocking propensity and the three network metrics (connectivity, strength, and closeness) using Bayesian phylogenetic linear models. Finally, we calculated Pagel’s λ to estimate the strength of the phylogenetic signal present in flocking propensity and the raw (i.e., untransformed) species-level network metrics, using the ‘phytools’ package (Revell 2024) propensity was mapped onto a consensus phylogenetic tree using the *contMap* function.

### Null models

To complement our primary analyses based on raw (observed) network metrics, which reflect a combination of population effects (e.g., species abundances) and structural processes (e.g., nuclearity), we implemented a null model approach to assess whether species-level patterns in network structure deviated from expectations under random assembly. Specifically, we generated 500 null models for each of the 83 locations (*n* = 41,500 null models) by randomizing species occurrences across flocks while preserving both row and column sums. This ensured that flock richness (i.e., number of species per flock), and species occurrence frequencies (i.e., number of times a species occurred in a flock) remained constant, thereby controlling for differences in flock size and species abundance, respectively. Each randomized flock × species matrix was then projected into a species × species co-occurrence matrix, from which we computed network metrics (connectivity, strength, and weighted closeness) for all species. For each species in each location, we calculated a *z*-score for each metric as

$$\begin{aligned}z-score& = (observed\,\,value - mean\,\, of\,\, null\,\, values)\\& / standard\,\,deviation\,\, of\,\, null \,\,values\end{aligned}$$

These *z*-scores quantify the degree to which observed network metrics differ from those expected under random species co-occurrence, given species’ local abundance and flock sizes. Finally, we used these *z*-scores as response variables in Bayesian phylogenetic linear models to assess the extent to which species’ phenotypic traits predicted their structural roles in flock networks.

## Results

**Fig. 1:**
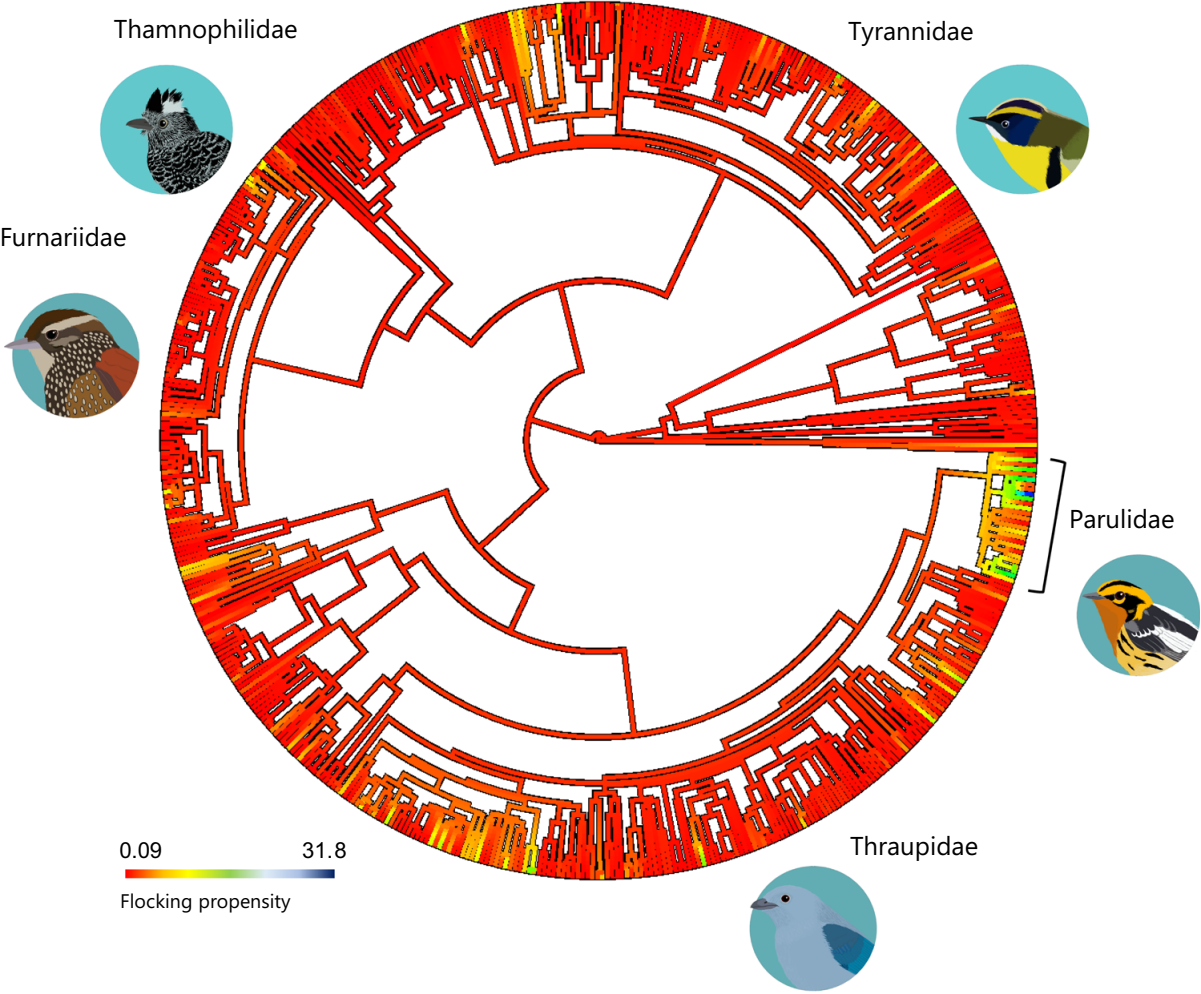
Phylogenetic mapping of the estimated average flocking propensity for 559 species based on data from 3,458 species-mixed bird flocks. Ancestral characters at internal nodes were estimated using maximum likelihood. The families with the highest flocking propensity estimates are highlighted

Species-level network metrics and flocking propensity showed a moderate and significant phylogenetic signal (*flocking propensity*: λ = 0.316, *p* < 0.001; *connectivity*: λ = 0.266,* p* < 0.01; *unweighted closeness*: λ = 0.153, *p* = 0.032; *strength*: λ = 0.173, *p* < 0.001). Tanagers (Thraupidae), New World flycatchers and allies (Tyrannidae), and ovenbirds and woodcreepers (Furnariidae) were the families with a greater number of flocking species (Table S2). Species’ propensity to join mixed-species bird flocks showed small variation within- and across families with the exception of the New World warblers (Parulidae), where this behaviour was remarkably heterogenous (range: 0.1–31,8%) (Fig. 1). Flocking propensity was strongly correlated with *connectivity* and *strength*, but not with *closeness* (Fig. S2). The three species-level network metrics were strongly correlated with each other (Fig. S2).

**Fig. 2:**
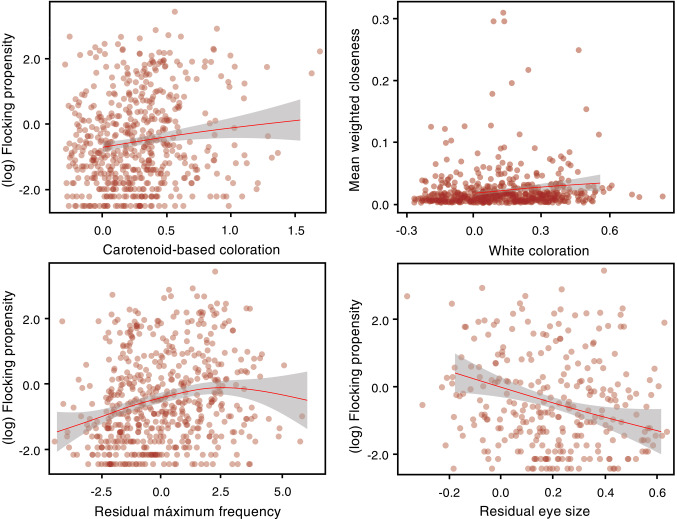
Relationship between flocking propensity and closeness, different avian traits related to sensorial capabilities. Each dot represents the average value for each species. Linear regressions were fitted using Generalized Additive Models (GAM)

As potential predictors of variation in flocking propensity and nuclearity measures, we quantified a range of morphological, visual and acoustic attributes for each species (see Methods). The strongest predictors of species’ propensity to join mixed-species bird flocks were (residual) maximum song frequency, (residual) eye size, and carotenoid-based coloration (Table 1), suggesting that species more prone to join mixed-species bird flocks emit vocalization at higher frequencies, exhibit a greater amount of carotenoid-based coloration, and tend to have relatively smaller eyes are (Fig. 2a-c; Fig. S3). Residual eye size was also the most relevant predictor of species connectivity (Table 1). Meanwhile, the amount of white plumage was a strong predictor of closeness (Table 1), which suggests that species with whiter plumages tend to occupy a more central position in the network (Fig. 2d). Lastly, interaction strength was positively correlated with the residual maximum frequency (Table 1).

**Table 1:** Predictors of flocking propensity and species-level network metrics in Neotropical birds (559 species). Mean effect sizes and 95% credible intervals (CI) computed with Bayesian phylogenetic models are shown. (^1^) This variable was analyzed using a subset (282 species)

	Mean	Lower	Upper
Flocking propensity			
Intercept	-2.417	-2.771	-2.058
*White*	0.349	-0.229	0.924
*Green*	0.104	-0.046	0.254
*Carotenoid-based*	0.236	0.044	0.427
*Melanin-based*	0.021	-0.147	0.186
*Beak shape*	0.037	-0.014	0.087
*Res. eye size*^*1*^	-0.612	-1.236	0.009
*Res. max frequency*	0.040	0.008	0.072
Connectivity (normalized degree)			
Intercept	0.004	-0.017	0.026
*White*	0.091	-0.173	0.355
*Green*	-0.013	-0.079	0.053
*Carotenoid-based*	-0.007	-0.093	0.077
*Melanin-based*	-0.054	-0.127	0.018
*Beak shape*	0.004	-0.017	0.026
*Res. eye size*^*1*^	-0.204	-0.506	0.091
*Res. max frequency*	-0.003	-0.017	0.010
Closeness			
Intercept	0.016	-0.000	0.032
*White*	0.036	0.002	0.070
*Green*	-0.003	-0.012	0.005
*Carotenoid-based*	0.002	-0.009	0.012
*Melanin-based*	-0.002	-0.012	0.006
*Beak shape*	0.001	-0.001	0.004
*Res. eye size*^*1*^	-0.031	-0.067	0.006
*Res. max frequency*	0.000	-0.001	0.002
Strength			
Intercept	0.569	0.042	1.081
*White*	0.574	-0.250	1.398
*Green*	0.107	-0.119	0.332
*Carotenoid-based*	-0.057	-0.347	0.229
*Melanin-based*	0.078	-0.164	0.326
*Beak shape*	-0.009	-0.084	0.065
*Res. eye size*^*1*^	-0.589	-1.445	0.262
*Res. max frequency*	0.059	0.011	0.107

We obtained quite similar results using PGLS; in most cases, the strongest predictors detected with the Bayesian models showed a statistically significant relationship with these models as well (Tables S3).

The null model approach confirmed the results obtained using the observed network metrics. We found that species with a higher number of connections and stronger than expected by chance tend to show a greater amount of white and carotenoid-based coloration and produce vocalizations at higher frequencies (Table 2; Table S4).

**Table 2:** Results of Bayesian phylogenetic linear models examining the relationship between *z*-scores of network metrics obtained using a null model approach and different phenotypic attributes for the full dataset (559 bird species), except for the variable residual eye size, which was analyzed using a subset (282 species)

	Mean	Lower	Upper
*z*-score connectivity			
Intercept	0.620	0.203	1.087
*White*	0.657	-0.185	1.496
*Green*	0.072	-0.161	0.305
*Carotenoid-based*	0.261	-0.031	0.550
*Melanin-based*	0.000	-0.237	0.231
*Beak shape*	-0.038	-0.110	0.035
*Res. eye size*	0.373	-0.979	1.704
*Res. max frequency*	0.047	0.000	0.095
*z*-score closeness			
Intercept	0.002	-0.635	0.677
*White*	0.682	-0.488	1.848
*Green*	-0.216	-0.575	0.141
*Carotenoid-based*	-0.073	-0.518	0.368
*Melanin-based*	0.006	-0.352	0.361
*Beak shape*	0.024	-0.089	0.138
*Res. eye size*	0.547	-0.235	1.328
*Res. max frequency*	-0.000	-0.075	0.075
*z*-score strength			
Intercept	0.238	-0.152	0.672
*White*	0.765	0.034	1.494
*Green*	-0.007	-0.207	0.191
*Carotenoid-based*	0.427	0.181	0.673
*Melanin-based*	-0.144	-0.350	0.059
*Beak shape*	-0.024	-0.087	0.039
*Res. eye size*	0.201	-0.506	0.991
*Res. max frequency*	0.005	-0.036	0.046

Although similar traits emerged as predictors in both analyses, their effects were associated with different network metrics depending on whether observed or null-model standardized values were considered (Tables 1 and 2).

## Discussion

We used an extensive dataset and a phylogenetic framework to explore the relationship between species’ flocking propensity, their connectivity, and position within mixed-species flock networks and phenotypic attributes. Although nuclear species are thought to enhance the cohesion of species-mixed flocks through the conspicuous nature of their vocalizations or coloration (Hutto 1994), this idea has not been formally tested at a macroevolutionary scale. By taking advantage of original data and recently published databases, we provide the first large-scale assessment of how visual and acoustic traits relate to species importance in the formation and maintenance of mixed-species flocks. Our results revealed that species with higher flocking propensity tend to have relatively smaller eyes, emit vocalizations at higher frequencies, and exhibit a greater amount of carotenoid-based coloration. In addition, we found that the amount of white plumage predicts a species’ centrality within the network. These findings, combined with the moderate phylogenetic signal detected in the species flocking propensity and analyzed network metrics, suggest that those species more prone to join mixed-species flocks and that play a nuclear role in them might present specific morphological and sensory adaptations.

The main benefit of joining mixed-species flocks lies in the transfer of information about predation risk and novel foraging opportunities through various communication pathways (Mangini et al. 2022; reviewed in Goodale et al. 2020). In this context, acoustic signaling plays a crucial role in flock formation and cohesion (Goodale and Kotagama 2005). For instance, Suzuki (2012) provided experimental evidence that long-distance calling by the willow tit (*Poecile montanus*) facilitates the establishment of mixed-species flocks. Species with distinct and less overlapping vocalizations are more likely to act as leaders or sentinels within their social groups, as they can effectively communicate warnings without masking or interfering with the alarm calls of others (Barbosa and Castellanos 2005). In the same vein, in a small comparative study, Pagani-Núñez et al. (2018) reported that leaders uttered less similar (i.e., more distinctive) calls than occasional species. Consequently, species whose vocalizations are less likely to overlap with others (such as those emitting at higher frequencies) may attain higher levels of nuclearity, acting as social hubs. Our results support this hypothesis: we found that species that participate frequently and consistently in flocks (high flocking propensity; high interaction or co-occurrence strength) tend to vocalize at higher frequencies, potentially avoiding acoustic interference and enhancing their detectability within the community (e.g., García-Navas et al. 2023). A similar pattern emerged when we restricted analyses to the three best-represented families: Thraupidae, Tyrannidae, and Furnariidae (Fig. S4). Regarding this latter, Beco et al. (2021) reported that male antwrens (Thamnophilidae) that forage in mixed-species flocks tended to have loud songs with higher peak frequencies and exhibit more contrasting upper parts (with brighter and more patterned wing coverts), which agrees with our findings at broader phylogenetic scale.

Regarding visual communication, many nuclear species are known for their conspicuous coloration, making them easily recognizable and attractive to other birds. For instance, brightly colored species such as tanagers (*Tangaras* spp.) may be more likely to attract other birds to join their flock. However, some studies have also shown that species with duller coloration (e.g., grey or brown plumages) can also act as nuclear species (e.g., Amaral and Ragusa-Netto 2008). In our analysis, we found that species with a higher proportion of white plumage exhibited greater closeness, indicating that these species occupy a more central position in flocking networks, which could be due to the fact that these species are more easily detected by others. On the other hand, species with a greater amount of carotenoid-based coloration were more prone to flock. This finding could have to do with the potential role of carotenoids as ‘honest’ signals of individual quality. Carotenoid-based coloration is costly to produce and maintain, as carotenoids must be obtained through the diet and play essential roles in physiological functions such as the immune system (Delhey et al. 2010). In social contexts like mixed-species flocks, carotenoid-rich plumage may serve as an honest signal of individual condition or resource-acquisition ability, both to conspecifics and heterospecific flock members (e.g., García-Navas et al. 2012). Although the deposition of carotenoids leads to showy and apparently conspicuous plumages, it has been suggested that some carotenoid-based colorations are quite cryptic against backgrounds of green leaves in closed environments, and hence this ornamentation could be optimized for crypsis (Delhey et al. 2010). Alternatively, visually conspicuous species may also be more detectable to potential predators and, consequently, these species might obtain disproportionate benefits from joining mixed-species flocks through mechanisms such as dilution effects or predator confusion (Stevens 2013).

Concerning antipredator strategies, the ‘many eyes’ hypothesis posits that larger groups enhance collective vigilance, improving predator detection (Morse 1977). Based on this, species with a greater tendency to join mixed flocks and play a more nuclear role in them may have a less developed visual system (i.e., smaller eyes). We found support for this prediction as our results revealed the existence of a strong relationship between residual eye size, a morphological proxy for visual acuity (Caves et al. 2024), and flocking propensity and connectivity. Species with lower relative eye size (smaller eyes than expected based on their body size) tended to flock more frequently and have more connections. This finding suggests that the evolution of eye morphology in birds is may be shaped by the risk of predation. This is consistent with findings by Beauchamp (2023) in a study including species that form flocks regularly or occasionally and solitary species (*n* = 660 species). Beauchamp (2023) reported that solitary species, which cannot rely on group vigilance, have larger eyes than flocking species. Our results reinforce the idea that species that do not benefit as often from the surveillance exercised by others may have evolved to rely more heavily on their own visual system for tasks like hunting or detecting potential threats. This could lead to more developed visual acuity or wider fields of vision.

Lastly, we found no evidence for a non-linear, asymptotic pattern in which generalist species with intermediate traits (“jack-of-all-trades” beak morphology) occupy central positions in the network and maintain more connections. This suggests that trophic generalism, as captured by beak shape, may not be a major driver shaping species associations in mixed-species flocks. Instead, flock composition may be structured more strongly by behavioural traits or by traits directly mediating resource use, such as diet (García-Navas and Martín del Campo, *submitted*).

Overall, our study revealed a link between species’ topological importance in heterospecific association networks and certain phenotypic attributes. These associations contribute to explain the moderate conservatism observed in the metrics employed to assess species’ positional importance. Thus, certain species play a nuclear role in flocks, irrespective of the details of the communities in which they occur. In this sense, the structural function of a species within the network can be viewed as a behavioral trait with potential evolutionary significance. This perspective opens avenues for understanding social roles not merely as emergent properties of ecological contexts, but as evolved traits potentially shaped by natural selection. If certain phenotypic traits consistently predict a species’ connectivity and position in flocking networks across environments and regions, this would imply that topological positioning confers fitness advantage, such as enhanced protection from predators, increased access to information, or improved foraging efficiency (Mangini et al. 2023). Consequently, species nuclearity in heterospecific or conspecific networks may be subject to selective pressures analogous to those acting on morphological or physiological traits (Oh and Badyaev 2010). Future comparative work integrating fitness outcomes and social network roles across species and lineages will be essential to determine whether centrality-related traits represent adaptations or evolutionary byproducts of other ecological processes.

## Supplementary Information

Below is the link to the electronic supplementary material.

Supplementary file1 (DOCX 4268 KB)

## Data Availability

All associated data, metadata and code can be found on Dryad [10.5061/dryad.vx0k6dk4z]. Supplementary material is available online.

## Declarations

### Declaration of AI use

We have not used AI-assisted technologies in creating this article.
