# Correction: Acoustic and visual traits predict species nuclearity in Neotropical mixed-species bird flocks

**DOI:** 10.1007/s00442-026-05910-9

**Published:** 2026-05-28

**Authors:** Vicente García-Navas, Carlos Martínez-Nuñez

**Affiliations:** 1 Department of Ecology and Evolution, Estación Biológica de Doñana EBD (CSIC), Avenida, Américo Vespucio, 26, E-41092 Seville, Spain; 2 Department of Evolutionary Biology and Environmental Studies, University of Zurich, Zurich, Switzerland

**Correction: Oecologia (2026) 208:65**

10.1007/s00442-026-05900-x

In this article the caption to Fig. 1 was inadvertently updated with error; the correct version should have appeared as shown below.

Fig. 1 Phylogenetic mapping of the estimated average flocking propensity for 559 species based on data from 3,458 species-mixed bird flocks. Ancestral characters at internal nodes were estimated using maxi-mum likelihood. The families with the highest flocking propensity estimates are highlighted.

The original article has been corrected.

