# Supplementary material for: Acoustic and visual traits predict species nuclearity in Neotropical mixed-species bird flocks
Source: Oecologia. 2026 May 11;208(5):65. doi: 10.1007/s00442-026-05900-x (PMC13161015; doi:10.1007/s00442-026-05900-x)

**Supplementary Material**

**Acoustic and visual traits predict species nuclearity in Neotropical mixed-species bird flocks**

**Table S1.** List of ornithological studies from which information on the composition of species-mixed flocks was obtained.

| *N* flocks | Location | Data source |
| --- | --- | --- |
| 5 | Argentina | Ippi & Trejo (2003) |
| 33 | Argentina | Zarco et al. (2019) |
| 108 | Argentina | Mangini (2018) |
| 344 | Argentina | Fanjul et al. (2015) |
| 11 | Argentina-Brazil | Areta et al. (2013) |
| 1 | Bolivia | Remsen & Ridgely (1980) |
| 24 | Bolivia | Martínez (2003) |
| 91 | Bolivia | Matthysen et al. (2008) |
| 355 | Bolivia | Montaño-Centellas (2020) |
| 3 | Brazil | Della-Flora et al. (2013) |
| 3 | Brazil | Davis (1946) |
| 7 | Brazil | Guimarães & Guilherme (2021) |
| 1 | Chile | Vuilleumier (1994) |
| 59 | Chile | Medrano et al. (2020) |
| 60 | Colombia | Marín-Gómez & Arbeláez-Cortés (2015) |
| 64 | Colombia | Bohórquez (2003) |
| 111 | Colombia | Arbeláez-Cortés et al. (2011, 2012) |
| 276 | Colombia | Colorado et al. (2015) |
| 429 | Colombia | Jones & Robinson (2021) |
| 440 | Colombia | McDermott & Rodewald (2014) |
| 2 | Ecuador | Bonaccorso et al. (2004) |
| 2 | Ecuador | Poulsen (1996) |
| 46 | Ecuador | Guevara et al. (2011) |
| 51 | Ecuador | Rodewald & Rodewald (2003) |
| 228 | Ecuador | Vásquez-Ávila et al. (2021) |
| 5 | Paraguay | Hayes & Areco de Medina (1988) |
| 1 | Peru | Munn & Terborgh (1979) |
| 2 | Peru | Parra et al. (2019) |
| 24 | Peru | Astudillo, *unpublished data*^1^ |
| 32 | Peru | Munn (1985) |
| 121 | Peru | Ausprey Newell, *unpublished data*^1^ |
| 172 | Peru | Muñoz (2016) |
| 347 | Venezuela | Newell et al. (2014) |

1. Data reported in Montaño-Centellas et al. (2023)

References:

Arbeláez-Cortés, E., Rodríguez-Correa, H.A. & Restrepo-Chica, M. (2011) Mixed bird flocks: patterns of activity and species composition in a region of the Central Andes of Colombia. *Revista Mexicana de Biodiversidad*, 82, 639-651.

Arbeláez-Cortés, E. & Marín-Gomez, O.H. (2012) The Composition of Mixed-Species Bird Flocks in Alto Quindío, Colombia. *The Wilson Journal of Ornithology*, 124: 572–580.

Areta, J.I., Bodrati, A., Thom, G., Rupp, A.E., Velázquez, M., Holzmann, I., Carrano, E., & Zimmermann, C.E. (2013) Natural history, distribution, and conservation of two nomadic *Sporophila* seedeaters specializing on bamboo in the Atlantic Forest. *Condor*, 115: 237-252.

Bohórquez, C.I. (2003) Mixed-species bird flocks in a montane cloud forest of Colombia. *Ornitologia Neotropical*, 14: 67-78.

Bonaccorso, E. (2004) Avifauna of a High Andean forest: Bosque Protector Cashca Totoras, Bolivar Province, Ecuador. *Ornitología Neotropical*, 15: 483-492.

Colorado, G.J. & Rodewald, A.D. (2015) Assembly patterns of mixed-species avian flocks in the Andes. *Journal of Animal Ecology*, 84: 386-395.

Davis, D.E. (1946) A seasonal analysis of mixed flocks of birds in Brazil. *Ecology*, 27: 168-181.

Della-Flora, F., Leal Melo, G., Sponchiado, J. & Cáceres, N.C. (2013) Association of the southern Amazon red squirrel *Urosciurus spadiceus* Olfers, 1818 with mixed-species bird flocks. *Mammalia*, 77: 113-117.

Fanjul, M.E., Echevarria, A.L. & Martínez, M.V. (2021) Estructura y composición de las bandadas mixtas de aves invernales a lo largo del gradiente latitudinal en las selvas montanas de las Yungas, Argentina. *Acta Zoológica Lilloana*, 65: 268-286.

Guevara, E.A., Valarezo, J.C., Onofa, A. & Cupuerán, F. (2011) Mixed-species flock composition in a Northwestern Ecuadorian cloud forest. *Ornitologia Neotropical*, 22: 379-386.

Guimarães, D.P., & Guilherme, E. (2021) Structure and home range size of mixed-species bird flocks in a bamboo forest in southwestern Amazonia. *Acta Ornithologica*, 56: 95-108.

Hayes, F.E., & Areco de Medina, F.E. (1988) Notes on the ecology of the avifauna of Chore, Department of San Pedro, Paraguay. *El Hornero*, 13: 59-70.

Ippi, S. & Trejo, A. (2003) Dinámica y estructura de bandadas mixtas de aves en un bosque de Lenga (*Nothofagus pumilio*) del Noroeste de la Patagonia Argentina. *Ornitología Neotropical*, 14: 353-362.

Jones, H.H. & Robinson, S.K. (2021) Vegetation structure drives mixed-species flock interaction strength and nuclear species roles. *Behavioral Ecology*, 32: 69-81.

Mangini, G. (2018) *Las bandadas mixtas de aves como estrategia: comportamiento y estacionalidad en la selva pedemontana de las Yungas Australes*. Tesis Doctoral. Universidad Nacional de Tucumán, Argentina.

Martínez, O. (2003) Composición por especies y uso de sustratos por las bandadas mixtas de aves en un bosque nublado andino de Bolivia. *Ecología en Bolivia*, 38: 99-119.

Marín-Gómez, O.H. & Arbeláez-Cortés, E. (2015) Variation on species composition and richness in mixed bird flocks along an altitudinal gradient in the Central Andes of Colombia. *Studies on Neotropical Fauna and Environment*, 50: 113-129.

Matthysen, E., Collet, F. & Cahill, J. (2008) Mixed flock composition and foraging behavior of insectivorous birds in undisturbed and disturbed fragments of High-Andean *Polylepis* woodland. *Ornitologia Neotropical*, 19: 403-416.

McDermott, M.E. & Rodewald, A.D. (2014) Conservation value of silvopastures to Neotropical migrants in Andean forest flocks. *Biological Conservation*, 175: 140-147.

Medrano, F., Vukasovic, M.A., Chiappe, R., & Estades, C.F. (2020) Composition and structure of bird flocks in a temperate forest of central Chile. *Revista Chilena de Ornitología*, 26: 33-36.

Montaño‐Centellas, F.A. (2020) Interaction networks of avian mixed‐species flocks along elevation in the tropical Andes. *Ecography*, 43: 930-942.

Montaño-Centellas, F.A., Muñoz, J., Mangini, G.G., Ausprey, I.J., Newell, F.L., Jones, H.H., Fanjul, E.M., Tinoco, B.A., Colorado, G.J., Cahill Jennifer, R.A., Arbeláez-Cortés, E., et al. (2023) Network structure of avian mixed-species flocks decays with elevation and latitude across the Andes. *Philosophical Transactions of the Royal Society of London B*, 378: 20220099.

Munn, C. A. (1985). *Permanent canopy and understory flocks in Amazonia: species composition and population density*. In: *Neotropical Ornithology*. Buckley, P.A., Foster, M.S., Morton, E.S., Ridgely, R.S., & Buckley, F.G. (Editors), *Ornithological Monographs*, 36: 683-712.

Munn, C.A., & Terborgh, J.W. (1979) Multi-species territoriality in Neotropical foraging flocks. *Condor*, 81: 338-347.

Muñoz, J. (2016) *The role of facilitation in the structure of tropical bird communities: a case study of mixed-species flocks*. Thesis Dissertation. University of British Columbia, Canada.

Newell, F.L., Beachy, T.-A., Rodewald, A.D., Rengifo, C.G., Ausprey, I.J. & Rodewald, P.G. (2014) Foraging behavior of migrant warblers in mixed-species flocks in Venezuelan shade coffee: interspecific differences, tree species selection, and effects of drought. *Journal of Field Ornithology*, 85: 134-151.

Parra, E., Riegner, M., Novoa, J., & Martínez, A.E. (2019) Observations of Rufous-vented Ground Cuckoo *Neomorphus geoffroyi* associating with mixed-species flocks. *Cotinga*, 41: 94-97.

Poulsen, B.O. (1996) Relationships between frequency of mixed-species flocks, weather and insect activity in a montane cloud forest in Ecuador. *Ibis*, 138: 466-470.

Remsen & Ridgely (1980) Additions to the avifauna of Bolivia. *Condor*, 82: 69-75.

Rodewald, A.D. & Rodewald, P.G. (2003) Mixed-species bird flocks in primary and regenerating montane forests in Ecuador. *Cotinga*, 19: 51-54.

Vásquez-Ávila, B., Knowlton, J.L., Espinosa, C.I. & Tinoco, B.A. (2021) Habitat alteration modifies the structure and function of mixed-species flocks in an Andean landscape. *Biotropica*, 53: 1153-1162.

Wood, S. (2023) *mgcv*: Mixed GAM Computation Vehicle with Automatic Smoothness Estimation. https://cran.r-project.org/web/packages/mgcv/index.html

Zarco, A., Cueto, V.R., Sagrario, M.C., & Marone, L. (2019) Effects of livestock grazing on flocks of seed-eating birds in the central Monte desert, Argentina. *Canadian Journal of Zoology*, 97: 606-611.

**Table S2.** Identity and average flocking propensity for the 28 avian families registered in the compiled dataset.

| **Family** | **Average flocking propensity** | **S.D.** | ***n*** |
| --- | --- | --- | --- |
| Thraupidae | 1,910 | 2,83 | 126 |
| Tyrannidae | 1,492 | 2,48 | 109 |
| Furnariidae | 1,268 | 2,26 | 87 |
| Thamnophilidae | 0,549 | 0,56 | 40 |
| Parulidae | 6,990 | 7,66 | 27 |
| Picidae | 1,205 | 1,87 | 22 |
| Passerellidae | 2,022 | 2,56 | 20 |
| Turdidae | 0,990 | 1,23 | 16 |
| Fringillidae | 1,382 | 2,03 | 15 |
| Troglodytidae | 0,873 | 1,28 | 13 |
| Tityridae | 1,643 | 2,52 | 12 |
| Cardinalidae | 1,835 | 2,95 | 11 |
| Vireonidae | 2,590 | 2,66 | 11 |
| Icteridae | 0,814 | 1,45 | 10 |
| Cotingidae | 1,062 | 1,02 | 6 |
| Capitonidae | 1,847 | 2,92 | 5 |
| Corvidae | 0,475 | 0,32 | 5 |
| Trogonidae | 0,527 | 0,56 | 5 |
| Pipridae | 0,290 | 0,21 | 4 |
| Ramphastidae | 0,217 | 0,11 | 4 |
| Bucconidae | 0,531 | 0,19 | 3 |
| Cuculidae | 2,374 | 3,79 | 3 |
| Galbulidae | 0,376 | -- | 1 |
| Grallariidae | 0,145 | -- | 1 |
| Mimidae | 0,087 | -- | 1 |
| Momotidae | 0,087 | -- | 1 |
| Polioptilidae | 2,664 | -- | 1 |
| Rhinocryptidae | 0,087 | -- | 1 |

**Table S3.** Results of Phylogenetic Generalized Linear Models (PGLS) examining the relationship between flocking propensity and species-level network metrics and different phenotypic attributes for the full dataset (559 bird species), except for the variable residual eye size, which was analyzed using a subset (282 species).

|  | Estimate | Std. Error | *t*-value | *p* |
| --- | --- | --- | --- | --- |
| **Flocking propensity** | | | | |
| intercept | -1.030 | 0.381 | -2.704 | 0.007 |
| *White* | 0.915 | 0.697 | 1.312 | 0.190 |
| *Green* | 0.251 | 0.175 | 1.435 | 0.151 |
| *Carotenoid-based* | 0.593 | 0.221 | 2.682 | 0.007** |
| *Melanin-based* | 0.082 | 0.192 | 0.428 | 0.669 |
| *Beak shape* | 0.086 | 0.056 | 1.529 | 0.127 |
| *Res. eye size* | -3.762 | 1.969 | -1.911 | 0.057¤ |
| *Res. max frequency* | 0.094 | 0.037 | 2.583 | 0.010* |
| **Connectivity (normalized degree)** | | | | |
| intercept | 0.448 | 0.048 | 9.34 | <0.001 |
| *White* | 0.166 | 0.127 | 1.322 | 0.187 |
| *Green* | -0.003 | 0.032 | -0.091 | 0.927 |
| *Carotenoid-based* | 0.001 | 0.040 | 0.008 | 0.994 |
| *Melanin-based* | -0.037 | 0.029 | -1.287 | 0.199 |
| *Beak shape* | 0.004 | 0.009 | 0.461 | 0.645 |
| *Res. eye size* | -0.181 | 0.137 | -1.313 | 0.190 |
| *Res. max frequency* | -0.004 | 0.006 | -0.678 | 0.498 |
| **Strength** | | | | |
| intercept | 0.525 | 0.250 | 2.097 | 0.036 |
| *White* | 0.757 | 0.462 | 1.638 | 0.101 |
| *Green* | 0.120 | 0.115 | 1.046 | 0.296 |
| *Carotenoid-based* | -0.022 | 0.145 | -0.149 | 0.882 |
| *Melanin-based* | 0.100 | 0.126 | 0.794 | 0.427 |
| *Beak shape* | -0.009 | 0.037 | -0.235 | 0.813 |
| *Res. eye size* | -0.676 | 0.465 | -1.453 | 0.147 |
| *Res. max frequency* | 0.059 | 0.024 | 2.452 | 0.014* |
| **Closeness** | | | | |
| intercept | 0.016 | 0.007 | 2.165 | 0.031 |
| *White* | 0.037 | 0.017 | 2.175 | 0.030* |
| *Green* | -0.003 | 0.004 | -0.792 | 0.428 |
| *Carotenoid-based* | 0.002 | 0.005 | 0.383 | 0.702 |
| *Melanin-based* | -0.002 | 0.004 | -0.441 | 0.659 |
| *Beak shape* | 0.001 | 0.001 | 0.914 | 0.361 |
| *Res. eye size* | -0.036 | 0.017 | -2.142 | 0.033* |
| *Res. max frequency* | 0.000 | 0.001 | 0.561 | 0.576 |

**Table S4.** Results of Phylogenetic Generalized Linear Models (PGLS) the relationship between *z*-scores of network metrics obtained using a null model approach and different phenotypic attributes for the full dataset (559 bird species), except for the variable residual eye size, which was analyzed using a subset (282 species).

|  | Estimate | Std. Error | *t* | *p*-value |
| --- | --- | --- | --- | --- |
| ***z*-score degree** | | | |  |
| Intercept | 0.498 | 0.176 | 2.837 | 0.004 |
| *White* | 0.830 | 0.460 | 1.804 | 0.071¤ |
| *Green* | 0.094 | 0.118 | 0.796 | 0.426 |
| *Carotenoid-based* | 0.303 | 0.146 | 2.073 | 0.038* |
| *Melanin-based* | 0.028 | 0.105 | 0.265 | 0.791 |
| *Beak shape* | -0.038 | 0.034 | -1.098 | 0.272 |
| *Res. eye size* | 0.682 | 0.419 | 1.629 | 0.104 |
| *Res. max frequency* | 0.049 | 0.034 | 2.237 | 0.025* |
| ***z*-score closeness** | | | |  |
| intercept | -0.159 | 0.330 | -0.481 | 0.630 |
| *White* | 1.084 | 0.734 | 1.476 | 0.140 |
| *Green* | -0.195 | 0.185 | -1.053 | 0.293 |
| *Carotenoid-based* | -0.038 | 0.231 | -0.165 | 0.868 |
| *Melanin-based* | 0.063 | 0.187 | 0.338 | 0.735 |
| *Beak shape* | 0.018 | 0.056 | 0.321 | 0.748 |
| *Res. eye size* | 1.100 | 0.862 | 1.278 | 0.202 |
| *Res. max frequency* | 0.049 | 0.022 | 2.237 | 0.978 |
| ***z*-score strength** | | | |  |
| intercept | 0.088 | 0.145 | 0.605 | 0.545 |
| *White* | 0.974 | 0.381 | 2.559 | 0.011* |
| *Green* | 0.039 | 0.097 | 0.401 | 0.688 |
| *Carotenoid-based* | 0.434 | 0.121 | 3.598 | <0.001*** |
| *Melanin-based* | -0.106 | 0.087 | -1.223 | 0.221 |
| *Beak shape* | 0.014 | 0.014 | 1.053 | 0.292 |
| *Res. eye size* | -0.181 | 0.137 | -1.313 | 0.190 |
| *Res. max frequency* | 0.010 | 0.018 | 0.543 | 0.587 |

**Figure S1.** Examples of species co-occurrence networks derived from mixed-species bird flocks at two sites in Colombia: (*top*) Patasola Natural Reserve and (*bottom*) Finca Estrella de Agua. In each network, nodes represent bird species and edges indicate co-occurrence within mixed-species flocks. Node size is proportional to the number of connections (degree) of each species, such that larger nodes correspond to species that co-occur with a greater number of flock participants. Edge thickness reflects the frequency of co-occurrence between species, with thicker edges representing stronger associations. Networks were constructed from flock composition data compiled for each locality. These examples illustrate the structure of local flock networks from which species-level metrics (connectivity, strength, and closeness) were calculated. The two networks were selected to illustrate variation in network size and complexity among sites. Node positions were determined using a Fruchterman–Reingold force-directed layout. Photographs of some of the most nuclear (i.e., highly connected) species in each network are shown alongside the corresponding network to illustrate examples of species occupying prominent positions within the local flock structure. Photo credits: (a-c) Félix Uribe, (d) Joseph C. Boone, (e) Francesco Veronesi. All pictures are under Creative Commons licenses.

**
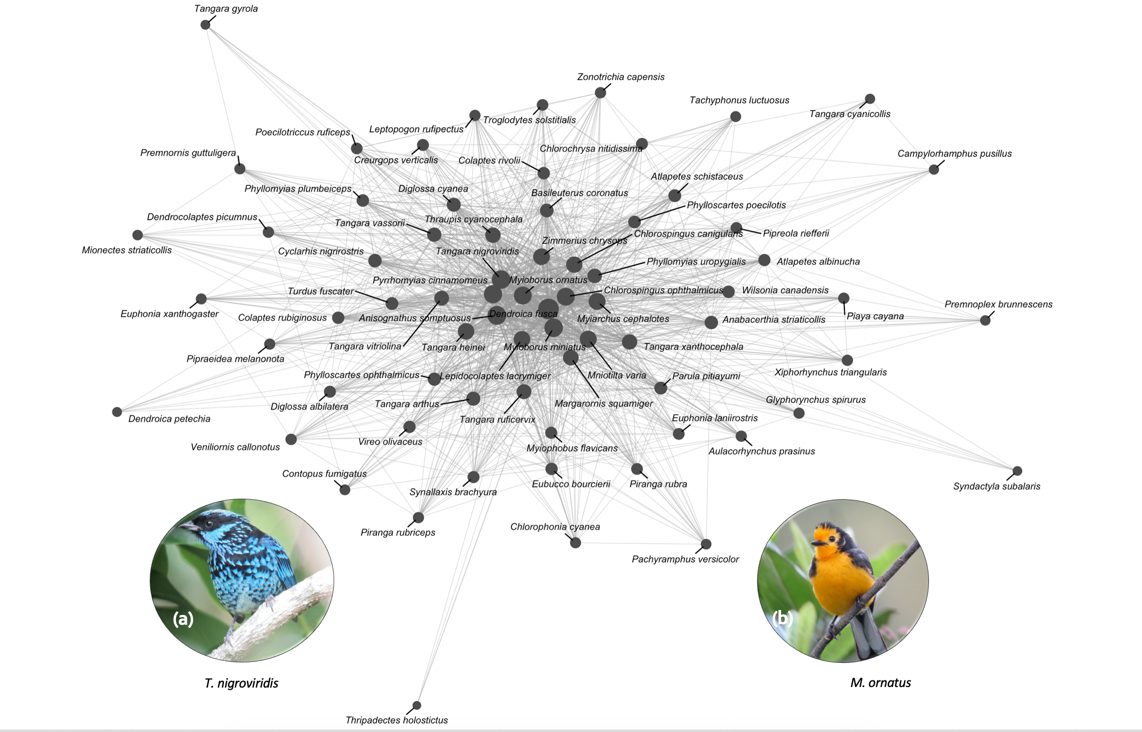
**

**
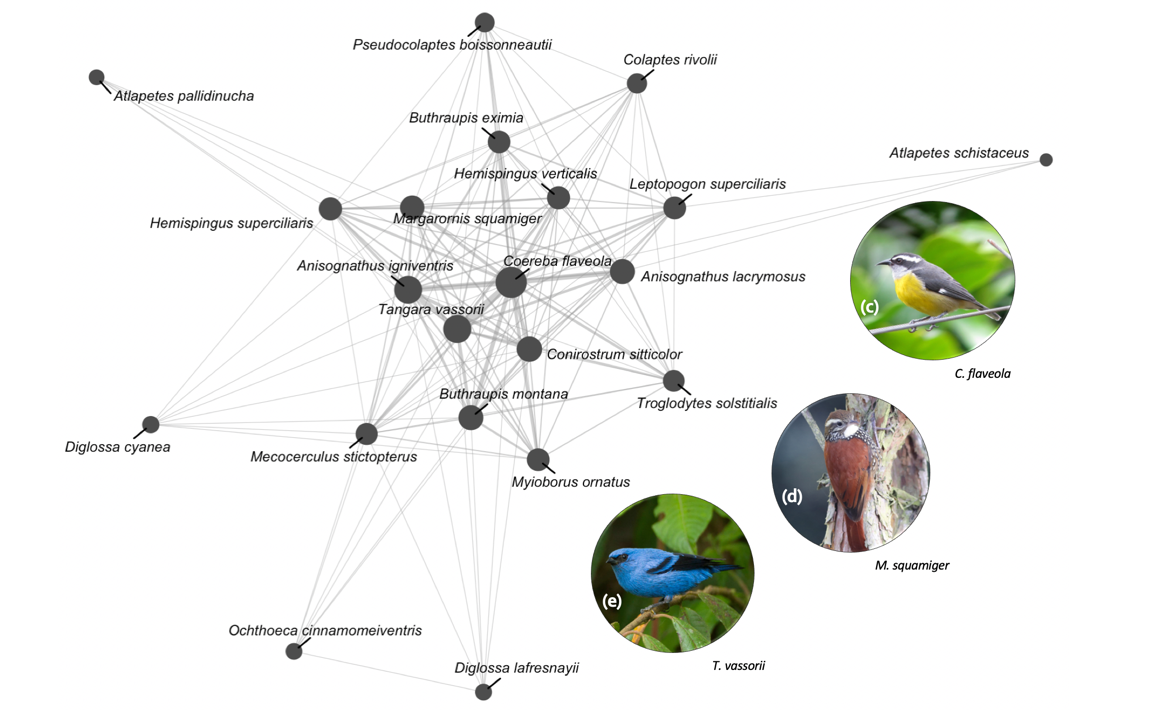
**

**Figure S2.** Association matrix to visualize the relationship between each pair of metrics. Estimates and *p*-values (*** = *p*<0.001; ** = *p*<0.01; * = *p*<0.05) obtained using PGLS are shown.


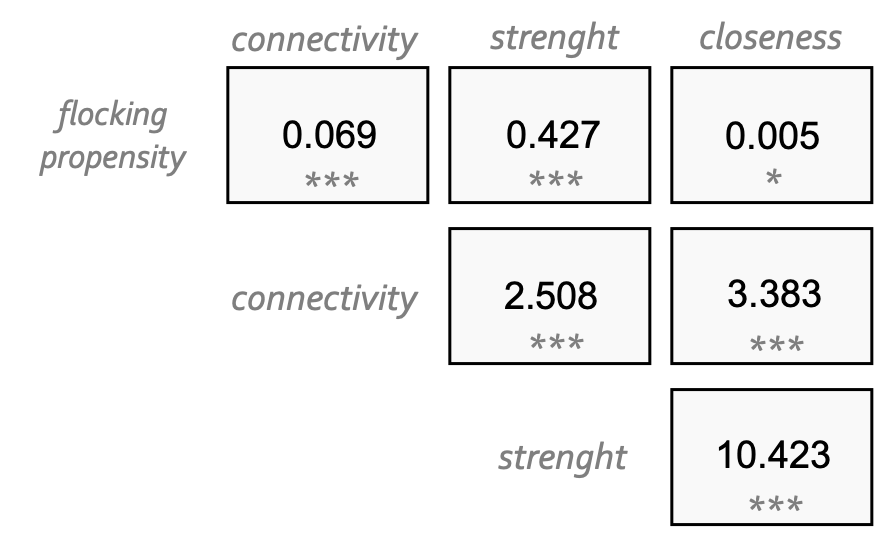


**Figure S3.** Examples of Neotropical bird species with relatively small eye size (left) and relatively large eye size (right). Species with a relatively smaller eye size tend to exhibit a higher flocking propensity. The observed flocking propensity is indicated in the blue circles. Photo credits: (a) Nigel Voaden, (b) Thibaut Aronson, (c) Joseph C. Boone, (d) Hector Bottai, (e) Charles J. Sharp, (f) Dario Sanches. All pictures are under Creative Commons licenses.


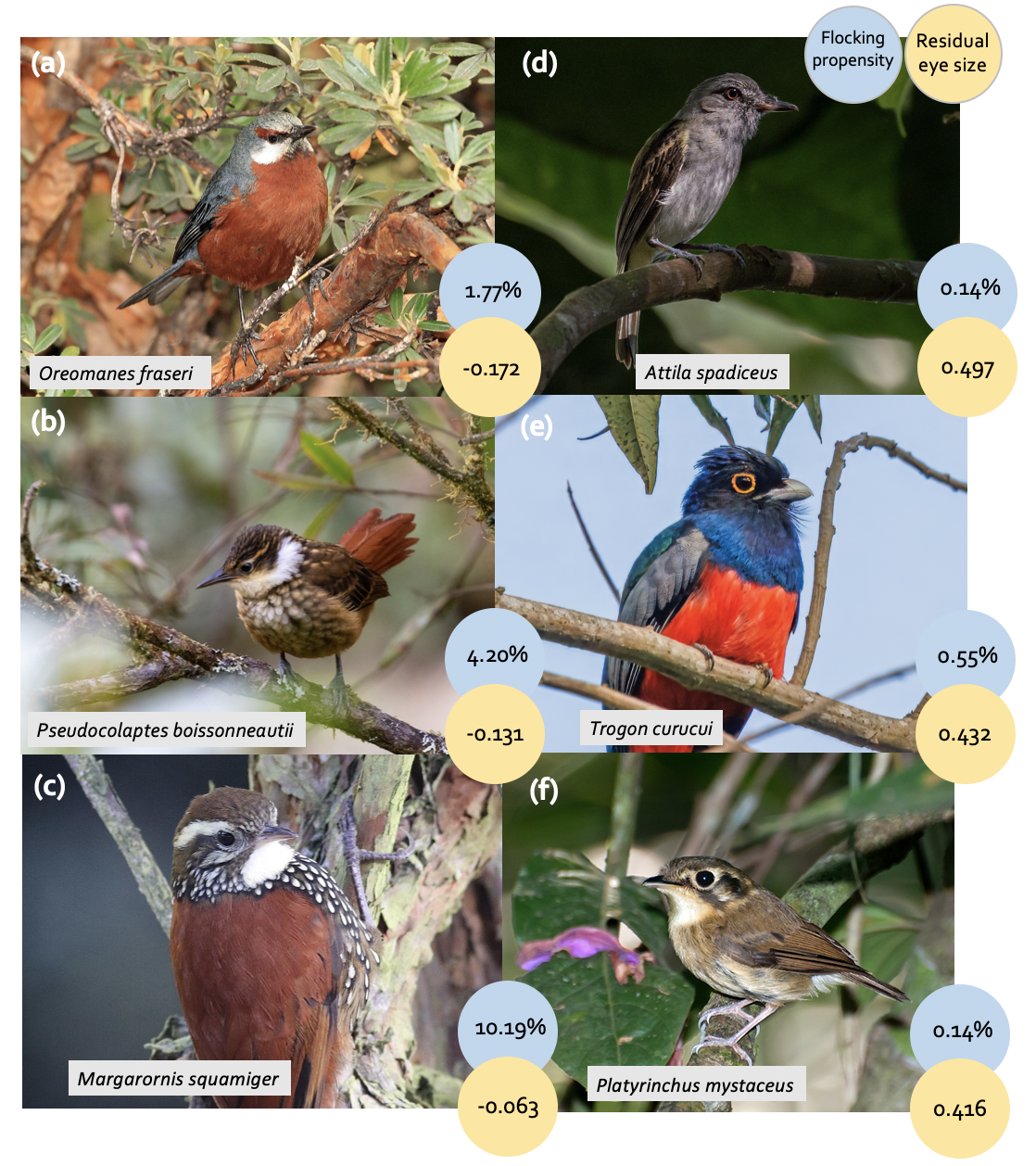


**Figure S4.** Relationship between flocking propensity and residual maximum frequency in: (a) tanagers (Thraupidae) (*n* = 126), (b) New World flycatchers and allies (Tyrannidae) (*n* = 109), and (c) ovenbirds and woodcreepers (Furnariidae) (*n* = 87).


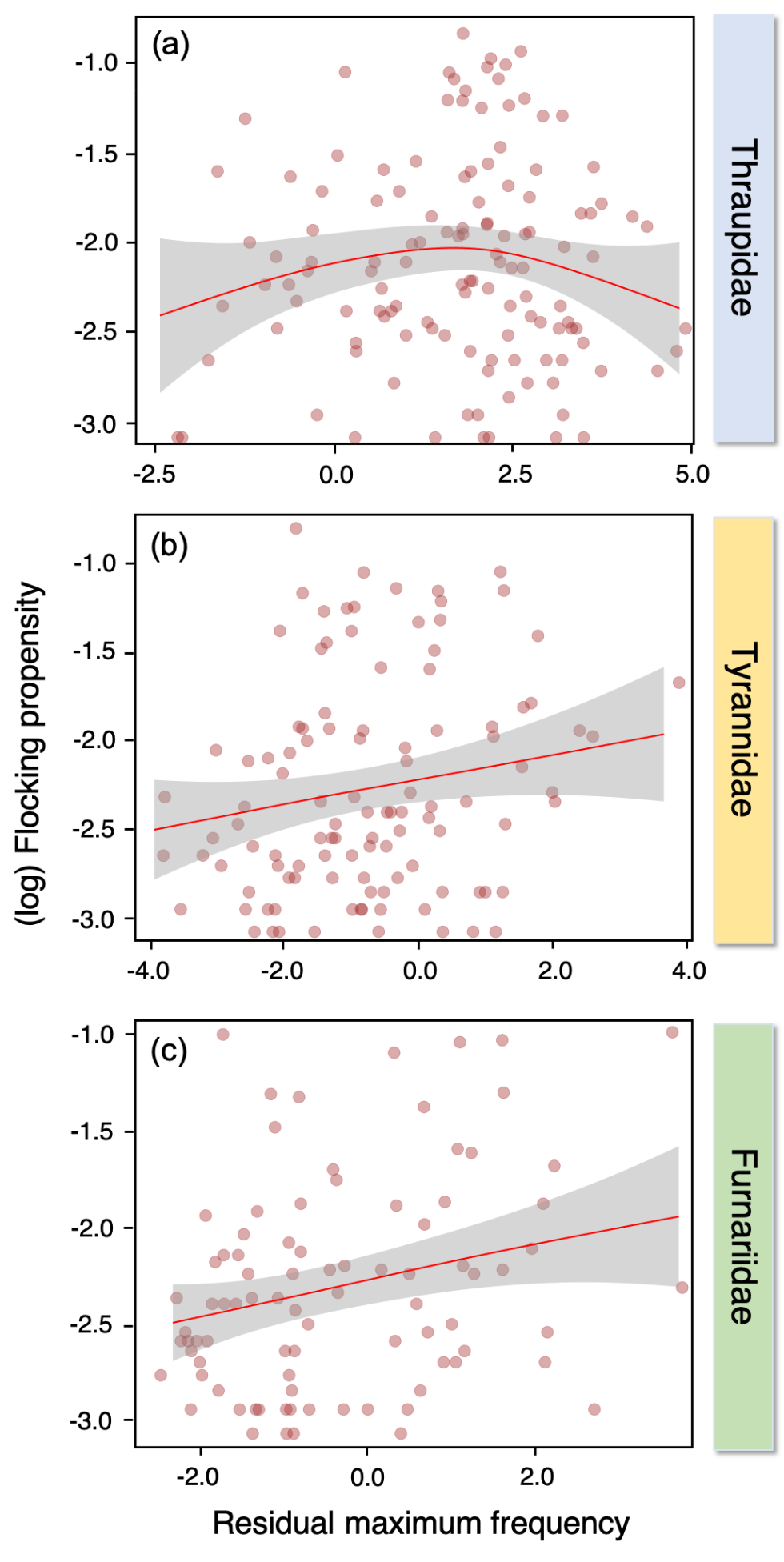

Supplement: Supplementary file 1 — Supplementary file1 (DOCX 4268 KB) [file 442_2026_5900_MOESM1_ESM.docx]
